# An Increased Risk of Reversible Dementia May Occur After Zolpidem Derivative Use in the Elderly Population: A Population-Based Case-Control Study: Erratum

**DOI:** 10.1097/01.md.0000466909.42832.c7

**Published:** 2016-06-19

In the article “An Increased Risk of Reversible Dementia May Occur After Zolpidem Derivative Use in the Elderly Population: A Population-Based Case-Control Study”,^1^ which appeared in Volume 94, Issue 17 of *Medicine*, the affiliations footnote was incorrectly given. Also, in Figure 1 the term “Normal Dementia” should have read “Non-Alzheimer Group”. The article has since been corrected online.
